# Non-Destructive Near-Infrared Technology for Efficient Cannabinoid Analysis in Cannabis Inflorescences

**DOI:** 10.3390/plants13060833

**Published:** 2024-03-14

**Authors:** Hamza Rafiq, Jens Hartung, Torsten Schober, Maximilian M. Vogt, Dániel Árpád Carrera, Michael Ruckle, Simone Graeff-Hönninger

**Affiliations:** 1 Department of Agronomy, Institute of Crop Science, University of Hohenheim, 70599 Stuttgart, Germany; 2 Biostatistics Unit, Institute of Crop Science, University of Hohenheim, 70599 Stuttgart, Germany; 3 Puregene AG, 4314 Zeiningen, Switzerland

**Keywords:** cannabinoid, near-infrared, partial least-squares regression

## Abstract

In the evolving field of cannabis research, scholars are exploring innovative methods to quantify cannabinoids rapidly and non-destructively. This study evaluates the effectiveness of a hand-held near-infrared (NIR) device for quantifying total cannabidiol (total CBD), total delta-9-tetrahydrocannabinol (total THC), and total cannabigerol (total CBG) in whole cannabis inflorescences. Employing pre-processing techniques, including standard normal variate (SNV) and Savitzky–Golay (SG) smoothing, we aim to optimize the portable NIR technology for rapid and non-destructive cannabinoid analysis. A partial least-squares regression (PLSR) model was utilized to predict cannabinoid concentration based on NIR spectra. The results indicated that SNV pre-processing exhibited superior performance in predicting total CBD concentration, yielding the lowest root mean square error of prediction (RMSEP) of 2.228 and the highest coefficient of determination for prediction (R^2^P) of 0.792. The ratio of performance to deviation (RPD) for total CBD was highest (2.195) with SNV. In contrast, raw data exhibited the least accurate predictions for total THC, with an R^2^P of 0.812, an RPD of 2.306, and an RMSEP of 1.651. Notably, total CBG prediction showed unique characteristics, with raw data yielding the highest R^2^P of 0.806. SNV pre-processing emerges as a robust method for precise total CBD quantification, offering valuable insights into the optimization of a hand-held NIR device for the rapid and non-destructive analysis of cannabinoid in whole inflorescence samples. These findings contribute to ongoing efforts in developing portable and efficient technologies for cannabinoid analysis, addressing the increasing demand for quick and accurate assessment methods in cannabis cultivation, pharmaceuticals, and regulatory compliance.

## 1. Introduction

*Cannabis sativa* L., belonging to the Cannabaceae family, possesses a noteworthy historical legacy as a versatile asset with significant contributions to multiple domains. Notably, it has been harnessed for medicinal purposes. Beyond its therapeutic applications, this plant finds utility in diverse industries, including food, textiles, and paper production [1]. Cannabidiol (CBD), delta-9-tetrahydrocannabinol (d9-THC), and cannabigerol (CBG) are important quality parameters in medicinal cannabis due to their significant medicinal effects. Renowned for its psychoactive properties and pain-relieving effects, THC plays a crucial role in managing conditions such as chronic pain and nausea [2]. On the other hand, CBD has gained attention for its potential anti-inflammatory, anticonvulsant, and anxiolytic properties, making it valuable in treating epilepsy, anxiety disorders, and inflammation-related conditions [3,4]. Although present in smaller quantities, CBG is recognized as a precursor to other cannabinoids and demonstrates neuroprotective and potential anti-inflammatory effects, making it an emerging target for research and medicinal applications [5,6].

The variability of phytocannabinoid content in cannabis inflorescences underscores the importance of reliable analytical methods for accurate and consistent quantification. Cannabis (*Cannabis sativa* L.) exhibits considerable genetic diversity, leading to variations in cannabinoid profiles among different genotypes and even within the same genotype grown under different conditions [7]. This inherent variability poses challenges in determining the precise concentration of cannabinoid, which is crucial for assessing the potency and potential medicinal effects of cannabis products. Traditional methods, such as high-performance liquid chromatography (HPLC) and gas chromatography (GC), have been widely employed for the analysis of cannabinoid concentration. However, these methods often require lengthy sample preparation and the use of hazardous solvents and are highly expensive, and time consuming [8]. This has led to the exploration of alternative techniques like near-infrared (NIR) spectroscopy, which offers a non-destructive and rapid approach to cannabinoid analysis, reducing the reliance on solvents and simplifying the overall process [9].

In recent years, NIR spectroscopy has emerged as a powerful tool for the comprehensive analysis of cannabis, covering aspects such as moisture content [10], growth stages [11], cannabinoids [12,13], and terpenes [14]. The analysis of NIR spectra entails comparing data with a reference method such as HPLC to construct predictive models employing statistical methodologies [15]. To predict the cannabinoids of different cannabis cultivars accurately, it is essential to have access to extensive and diverse sample sets that adequately represent the genetic and corresponding chemotypic diversity [16]. While existing methods often involve scanning ground or semi-ground inflorescence samples, a limited number of studies have explored the potential of NIR spectroscopy on whole inflorescence samples [10,13]. Although screening whole inflorescences presents a more time-efficient approach compared to scanning ground inflorescence methods, research in this domain has been constrained by small sample sizes and low variation in cannabinoid content [12].

The selection of a partial least-squares regression (PLSR) model, coupled with specific pre-processing techniques, is a critical aspect to be considered in the development of prediction models [17]. Standard normal variate (SNV) is instrumental in mitigating unwanted variations, such as baseline shifts and sample thickness differences, thereby ensuring the consistency and reliability of the spectral data [18]. Additionally, Savitzky–Golay (SG) smoothing reduced random noise while preserving the essential spectral features to improve data quality and facilitate more precise spectral interpretation [19]. Often, prediction models employ a comprehensive package or a combination of multiple techniques. In this study, we assessed the efficacy of pre-processing techniques, SNV and SG smoothing, individually, and compared them with the unprocessed raw data. This comparative analysis aims to find the individual impact of these techniques on predictive performance and their contributions to the overall modeling process.

The hypothesis tested in this study is that a hand-held NIR device in conjunction with pre-processing techniques such as SNV and SG smoothing can accurately quantify the concentrations of total CBD, total THC, and total CBG in whole cannabis inflorescences. The study aims to compare NIR measurements with reference values to assess the accuracy and reliability of portable NIR technology for cannabinoid analysis.

## 2. Results and Discussion

### 2.1. Quantitative Analysis Using HPLC

In this study, we analyzed a diverse set of cannabis genotypes, each exhibiting varying concentrations of total cannabidiol (total CBD), total delta-9-tetrahydrocannabinol (total THC), and total cannabigerol (total CBG). Our dataset comprised 890 genotypes encompassing a wide spectrum of cannabinoid concentration, ranging from zero to a maximum of 22.16% for total CBD, 16.33% for total THC, and 13.76% for total CBG. Among the three cannabinoids, total CBD exhibited the highest mean concentration at 7.40%, with a standard deviation (SD) of 5.12% (Table 1). Samples with diverse concentrations of cannabinoid led to an impartial population, enabling the model to make predictions without overfitting [13]. Therefore, this study is the most comprehensive and largest of its kind (Table 1), leading to the development of the most robust cannabinoid prediction model currently available.

### 2.2. Pre-Processing Techniques for NIR Spectra

**Figure 1:**
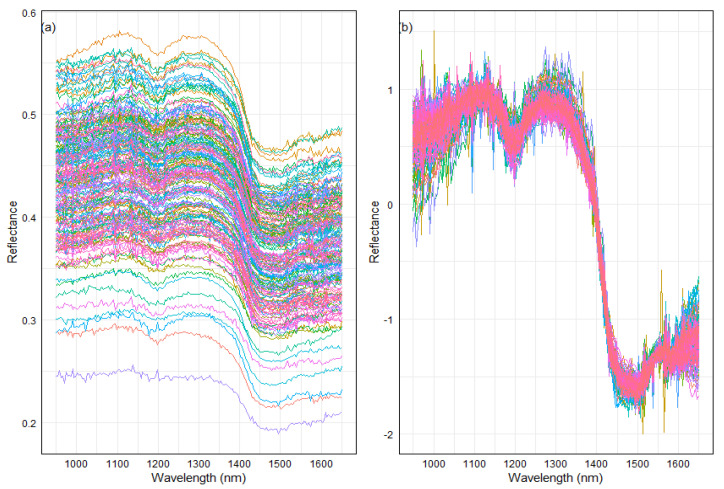
Near-infrared spectra of 200 genotype samples within the wavelength range of 950 to 1650 nm. The figure features two subplots, (**a**) raw spectra and (**b**) standard normal variate (SNV) spectra processing. In the raw data plot, each color spectrum represents the spectral signature across the wavelength range per sample. On the other hand, in the SNV plot, the spectra have been processed using standard normal variate transformation, resulting in a distinct representation of the spectral data.

In the context of cannabinoid prediction using near-infrared (NIR) spectroscopy, pre-processing techniques, such as standard normal variate (SNV) and Savitzky–Golay (SG) smoothing, are commonly employed together to enhance the efficacy of predictive models [13,20]. However, in a recent investigation [8], the efficiency of raw data and pre-processing techniques were examined through individual comparisons. Their study focused on 35 hemp samples in semi-fine powder for the prediction of total CBD and THC concentrations [8]. Nevertheless, to the best of our knowledge, whole inflorescences have not been investigated for the individual comparison of pre-processing techniques and raw data for the prediction of cannabinoid concentration using NIR spectroscopy (Figure 1).

### 2.3. PLSR Modeling for the Prediction of Total CBD, Total THC, and Total CBG Concentrations

The partial least-squares regression (PLSR) model is based on training (70%), data scaling, and cross-validation, ensuring a consistent and reproducible approach for assessing predictive accuracy. The predictive performances of the PLSR models for total CBD, total THC, and total CBG concentrations were compared between raw data, SNV, and SG smoothing (Table 2).

**Figure 2:**
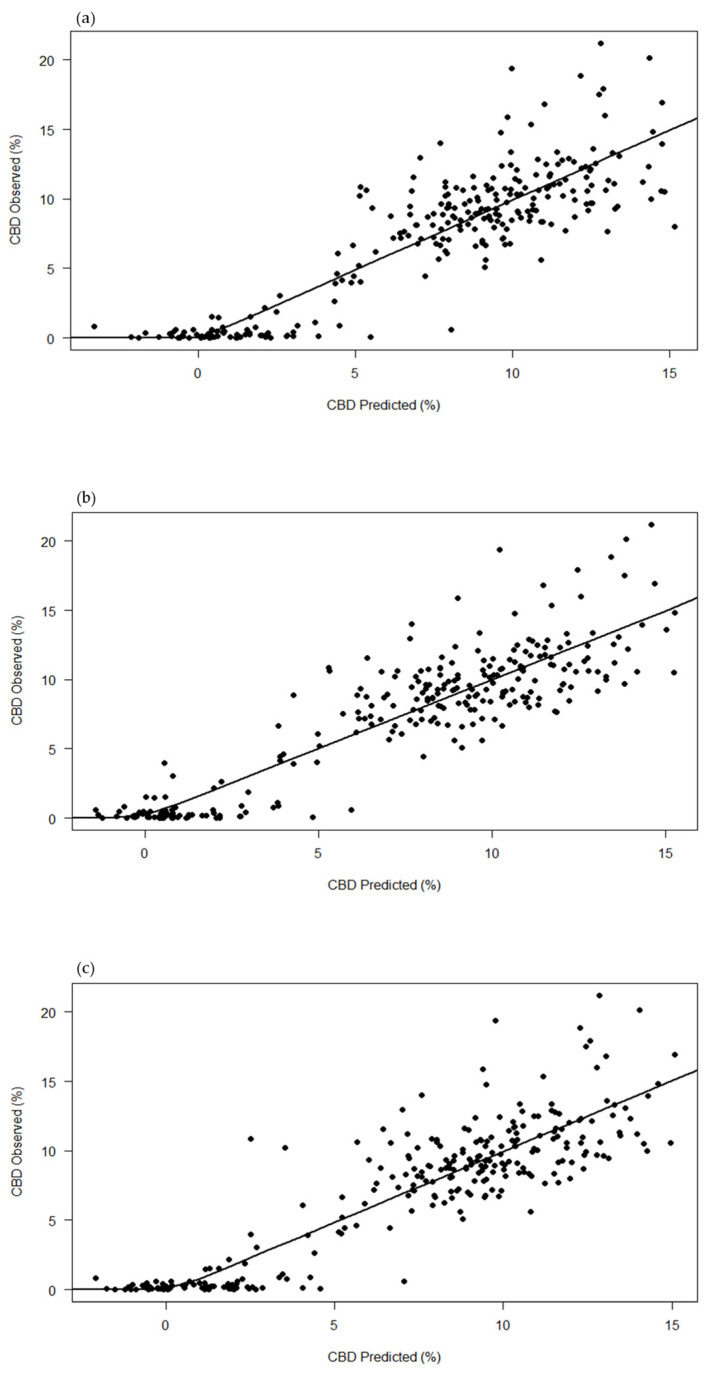
Observed versus predicted plot of the total cannabidiol (total CBD) concentration using the partial least-squares regression (PLSR) model. The plot compares the performance of three pre-processing techniques: (**a**) raw data, (**b**) standard normal variate (SNV), and (**c**) Savitzky–Golay (SG) smoothing. Each point represents an individual measurement, with the *x*-axis showing the predicted total CBD concentration and the *y*-axis showing the observed total CBD concentration.

In Table 2, the performance of different pre-processing techniques in predicting total CBD concentration is detailed. The application of standard normal variate (SNV) pre-processing exhibited superior performance, as evidenced by the lowest root mean square error of prediction (RMSEP) of 2.228 and the highest coefficient of determination for prediction (R^2^P) of 0.792. Additionally, the ratio of performance to deviation (RPD) for total CBD was highest (2.195) with SNV, indicating its robustness in accurately predicting CBD concentration. In contrast, the raw data resulted in the highest RMSEP (2.379) and the lowest R^2^P (0.763) and RPD (2.055) compared to both pre-processed datasets. Figure 2 visually represents the comparison of these results, showing that the predicted values with SNV (Figure 2b) are more closely aligned with the observed values, indicated by their proximity to the diagonal line, compared to raw data (Figure 2a) and SG smoothing (Figure 2c).

The PLSR model demonstrated optimal performance when coupled with SNV pre-processing for predicting total THC concentration (Table 2). This combination yielded the highest coefficient of determination for prediction (R^2^P) of 0.847 and ratio of performance to deviation (RPD) of 2.555, along with the lowest root mean square error of prediction (RMSEP) of 1.498. In contrast, both raw data and Savitzky–Golay (SG) smoothing techniques fell short in accuracy. Specifically, raw data resulted in an R^2^P of 0.812, an RPD of 2.306, and an RMSEP of 1.651, indicating less precise predictions compared to SNV pre-processing. These findings underscore the superiority of SNV pre-processing in enhancing the accuracy of total THC concentration predictions (Figure S1).

In predicting the concentration of total CBG, the results exhibited unique characteristics (Figure S1). Raw data produced the highest prediction correlation coefficient (R^2^P) of 0.806, accompanied by a ratio of performance to deviation (RPD) of 2.267 and a root mean square error of prediction (RMSEP) of 0.623. Notably, SNV pre-processing, which typically performed favorably for other cannabinoids in this study, yielded lower predictions with an R^2^P of 0.763, an RPD of 2.057, and an RMSEP of 0.687 (Table 2). Interestingly, SG smoothing demonstrated competitive performance comparable to the highest raw data prediction, achieving an R^2^P of 0.804, an RPD of 2.256, and an RMSEP of 0.627.

NIR spectroscopy has already been applied to the quantitative analysis of CBD [21], THC [13,21], and CBG [14] in fine powder [22], semi fine powder [8], and whole inflorescences [13,23]. Heterogeneity in the whole cannabis inflorescence presents a challenging analytic target for NIR spectroscopy [13] due to its sensitivity to both chemical and physical properties [24]. Therefore, a whole inflorescence with 10 spectra per sample was used in this study. In addition, a large sample size is pivotal in the training of NIR prediction to underline the spectral pattern more effectively [25], strengthen robustness to variations within the dataset [26], and mitigate the risk of overfitting [27]. In this investigation, we utilized a substantial sample size (*n* = 890), emphasizing the effectiveness of highlighting spectral patterns and dataset variations and reducing the risk of overfitting.

In the study conducted by [10], a predictive model was developed for the whole inflorescences, covering five cannabinoids within the wavelength range of 950 to 1650 nm. The PLSR model employed in their research demonstrated notable predictive accuracy, yielding the highest R^2^P of 0.89 for CBD concentration (SD = 1.84) and a contrasting R^2^P of 0.11 (SD = 0.20) for THC across a total of 194 samples [10]. For CBG concentration, which involved 187 whole inflorescence samples, the R^2^P was 0.43 (SD = 0.04). The lower variations observed in the CBG and THC datasets were identified as potential contributors to the lower prediction accuracy in their study [10]. In our investigation, where the total THC concentration exhibited substantial variation, with a standard deviation of 3.88 (Table 1), pre-processing with SNV resulted in the highest R^2^P of 0.847 (Table 2). Conversely, despite lower variations in total CBG concentration (SD = 1.34) compared to both total THC and total CBD concentrations (SD = 5.12), the raw data still yielded a noteworthy R^2^P of 0.806. Importantly, variation in cannabinoid concentrations alone does not translate into higher prediction accuracy. As previously mentioned [10], demonstrated that CBG concentration, characterized by extremely low variation (SD = 0.04), exhibited a higher R^2^P compared to THC (R^2^P = 0.11), which had relatively higher variation (SD = 0.20). Our study aligns with these findings, where total CBD concentration, marked by higher variation (Table 1), exhibited lower prediction accuracy in comparison to total THC and total CBG concentrations (Table 2).

In the comparison conducted by [13], low-cost NIRSG1 and mid-cost MicroNIR devices were evaluated on a total of 26 samples for THC concentration prediction using PLSR models, considering variations in whole inflorescences and different grinding levels. In their study, MicroNIR outperformed NIRSG1, achieving the highest prediction accuracy for whole inflorescences with an R^2^P of 0.93 and an RPD of 4.54, while NIRSG1 showed lower accuracy with R^2^P = 0.73 and RPD = 1.95 [13]. In our current study, we obtained the highest prediction accuracy for total THC concentration using the low-cost NIRSG1 device (R^2^P = 0.847 and RPD = 2.555). This was achieved through the application of the pre-processing SNV technique (Table 2). Notably, our research, conducted with a considerably larger sample size (*n* = 890), reinforces the robustness of our findings while utilizing the cost-effective NIRSG1 device.

The findings in this study underscore the critical influence of pre-processing techniques on the predictive performance of PLSR models for cannabinoid concentration. SNV pre-processing consistently produced superior prediction of both total CBD and total THC concentrations, emphasizing its effectiveness in reducing noise and enhancing the interpretability of spectral data. The raw data, while showcasing competitive prediction for CBG concentration, lagged when applied to total CBD and total THC concentration models. The unexpected outcome observed for the prediction of total CBG concentration requires further investigation. While SNV pre-processing was advantageous for other cannabinoids, it exhibited decreased performance for total CBG concentration. This suggests that the underlying spectral characteristics of total CBG may differ substantially from total CBD and total THC, necessitating alternative pre-processing strategies.

The reference values were obtained using high-performance liquid chromatography (HPLC), a widely trusted method. However, the findings from NIR spectroscopy, as indicated above, continue to hold relevance within the cannabis industry. Typically, only a single inflorescence (apical bud) per plant is utilized for cannabinoid analysis to represent the whole plant. Nevertheless, research studies have revealed significant variations in CBD concentrations among different inflorescences within the same plant [7,28]. For instance, a recent study [7], demonstrated significant differences in CBD concentration between top, mid, and low buds. To conduct a comprehensive and representative examination of the cannabis plant, multiple samples per plant are necessary, which can be highly expensive when using HPLC. Incorporating NIR spectroscopy alongside HPLC offers a robust, cost-effective, and representative approach that aligns with the standards of the cannabis industry.

The regulations that impose stringent limits on THC levels in cannabis genotypes, as seen in Europe with a threshold of 0.3%, present significant hurdles for the cultivation of non-psychoactive dominant varieties, such as CBD-dominant varieties. Considering these restrictions, breeding programs encounter substantial challenges when attempting to develop novel cultivars [29]. The risk of pollen contamination from a single THC-dominant plant can jeopardize the entire breeding initiative. NIR spectroscopy offers a potential solution by enabling comprehensive cannabinoid profiling at early growth stages [11]. This allows for a swift assessment to avoid potential contamination issues and aids in the early selection of relevant genotype profiles.

In conclusion, the results obtained in this study highlight the importance of tailoring pre-processing techniques to the specific cannabinoid of interest with an NIRSG1 device. The choice of pre-processing technique can significantly impact model accuracy and generalization; careful consideration should be given to the unique spectral characteristics of each compound. Further research is required with extended wavelength ranges to uncover the underlying factors contributing to the varying performance of pre-processing methods across different cannabinoids. Overall, this study demonstrates the versatility of PLSR modeling and the critical role of pre-processing in optimizing predictions for cannabinoid concentration with NIRSG1, offering valuable insights for researchers in the field of spectroscopy and analytical chemistry.

## 3. Materials and Methods

### 3.1. Sample Collection

In this study, cannabis plant samples were taken from Puregene AG’s breeding trials located in Zeiningen, Switzerland. A total of 890 unreplicated diverse cannabis genotypes grown at the same location during the year 2020 were included in our research. Each cannabis sample consisted of the whole apical inflorescence (top 15 cm), representing individual genotypes. To ensure consistency amongst samples, inflorescences were frozen shortly after harvest. The frozen material was then freeze dried, and dry inflorescences were closely monitored for humidity levels, ensuring humidity levels between 8% and 13%. Following this, the samples were stored in a dark environment, where the temperature remained consistently below 25 °C.

### 3.2. Near-Infrared (NIR) Spectroscopy

**Figure 3:**
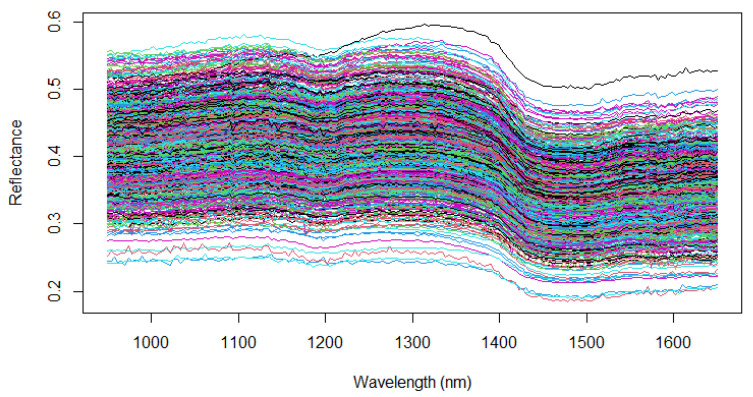
Raw near-infrared spectra of the 890 whole inflorescences with wavelength ranges from 950 to 1650 nm in the reflection mode. Each color spectrum represents the spectral signature across the wavelength range per sample.

Near-infrared spectra were acquired in the reflectance mode using a portable NIRSG1 device from Luxflux GmbH, Kusterdingen, Germany. The recorded wavelength ranged from 950 to 1650 nm with a 4 nm interval and a spectral resolution of 10 nm. Each scan took less than a second. To ensure comprehensive coverage of the samples, we measured each inflorescence ten times in the reflection mode. The spectrum used in the model for each plant was the average of these ten scans. To achieve more consistent spectra, each inflorescence underwent regular rotation during the measurement setups (Figure 3).

### 3.3. High-Performance Liquid Chromatography (HPLC)

Cannabinoid extractions from inflorescence material were performed through mechanical homogenization in a VWR Starbeater mill (VWR International, Radnor, PA, USA). Approximately 500 mg of plant inflorescence material (weight noted) and 15 mL of ethanol (99.6%, Ph.Eur. grade) were added to disposable 50 mL test tubes with zirconia beads (~2 mm diameter), and cannabinoid was extracted via shaking for 5 min at 25 Hz. An aliquot of the crude extract was directly filtered through a 0.2 µm PTFE syringe filter (or a 96 well format filter plate with 0.2 µm PTFE) and diluted as needed with ethanol.

The cannabinoid assay was run on a 1290 Infinity II Agilent HPLC system (Agilent Technologies, Santa Clara, CA, USA) equipped with a diode array detector (DAD), temperature-controlled column compartment, multisampler, and quaternary pump. The separation of the analytes was achieved on a Kinetex 1.7 µm EVO C18 100A 100 × 1.2 mm column (Phenomenex, Torrance, CA, USA). Full spectra were recorded from 200 to 400 nm, and absorbance at 230 nm was used to quantify cannabinoid content.

Instrument control, data acquisition, and integration were achieved with OpenLAB CDS 2.8 (Agilent Technologies, Santa Clara, CA, USA) software, applying an identification and quantification method based on an 8-level external standards calibration curve. To confirm the identity of analytes in the plant material, retention time and peak purity were compared with the signals acquired from certified reference materials (CRMs).

The calibration curve used for quantification of the most common cannabinoid was obtained by analyzing serial dilutions of cannabinoid mixtures produced in-house from commercially available cannabinoid CRMs. Namely, cannabidiol (CBD), cannabigerol (CBG), cannabidiolic acid (CBDA), cannabigerolic acid (CBGA), delta-9-tetrahydrocannabinol (d9-THC), and tetrahydrocannabinolic acid (THCA).

The concentration of cannabinoid is calculated as a percentage of the dry mass of cannabis inflorescence [% *w*/*w*]. Total CBD is calculated according to the following formula: CBD [% *w*/*w*] + CBDA × 0.877 [% *w*/*w*], with the factor of 0.877 accounting for decarboxylation of the CBDA molecule. Similarly, the formulas for the other relevant major cannabinoids are: total THC = d9-THC [% *w*/*w*] + d9-THCA × 0.877 [% *w*/*w*], total CBG = CBG [% *w*/*w*] + CBGA × 0.877 [% *w*/*w*]. The total cannabinoid concentration is calculated as the sum of the above total concentration values for a single cannabinoid.

### 3.4. Pre-Processing Techniques

Information of interest in the NIR spectra can be interfered with due to different factors such as spectra noise. Various mathematical pre-processing techniques can be used to deal with these interferences. In this study, wavelength information was used in three versions: as raw observed values, as smoothed values, and as standardized values. For SG smoothing operation, raw observed values $y_{ik}$ at the *i*-th wavelength within each spectrum *k* were smoothed with Equation (1).

(1) $$y_{s_{ik}}=\frac{1}{2m+1}\sum_{j=-m}^{m}c_{j}\cdot y_{\left(i+j\right)k}$$

where $y_{s_{ik}}$ is the smoothed value at wavelength *i* in spectrum *k*, *m* is 3, $c_{j}$ is the coefficients determined by the chosen parameters, and $y_{\left(i+j\right)k}$ denotes the (*i + j*)-th data points in spectrum *k* within the smoothing window centered around wavelength i. In our case, a polynomial of order 3 (*p* = 3) was chosen to capture data trends, with a derivative order of 0 (*d* = 0) focusing on data smoothing rather than derivative extraction. We used a window size of 7 (2*m* + 1 = 7) consecutive points during polynomial smoothing.

Standardized normal value (SNV) was applied row-wise to all observations within a spectrum in the dataset to normalize the data (Equation (2)). We extracted each spectrum as a row vector from the dataset, centered the data by subtracting the spectrum’s mean from each data point, and scaled it by dividing each data point by the spectrum’s standard deviation. This resulted in rows of normalized data with a centered baseline and reduced intensity variations.

(2) $$SNV\left(y_{ik}\right)=\left(y_{ik}-\mu_{k}\right)/\sigma_{k}$$

In Equation (2), $SNV\left(y_{ik}\right)$ represents the SNV normalized value of $y_{ik}$. The variable $\mu_{k}$ corresponds to the estimated mean of the data points $y_{ik}$ within the spectrum *k*, and $\sigma_{k}$ represents the estimated standard deviation of these data points. By applying this normalization technique row-wise to each spectrum in our dataset, we centered the baseline and reduced intensity variations, ensuring that the data remained on a consistent scale for subsequent analysis. Smoothing and normalization was performed for all *I* wavelengths and *K* spectra, where *I* and *K* are the number of wavelengths and spectra, respectively.

### 3.5. Partial Least-Squares Regression (PLSR)

Utilizing partial least-squares regression (PLSR), the relationship between the predictor variables (wavelength spectra) and the target response variable (total CBD, total THC, and total CBG) was modeled. Ten-fold cross-validation was employed to determine the optimal number of latent variables. The dataset was randomly divided into ten subsets, and the PLSR model was trained and validated iteratively. Equation (3) represents the PLSR model equation used in our analysis:

(3) T=X⋅W

where ***T*** represents the *K* × *A* matrix of orthogonal latent variables, ***X*** is a *I* × *K* matrix of wavelength $y_{ik}$, and ***W*** is a *K* × *A* matrix of weights. The matrix ***T*** has the property that errors in ***X*** = ***TP*** + ***E*** and ***Y*** = ***TC*** + ***F*** are small [30,31], where ***Y*** is the vector of the response variable, ***P*** and ***C*** are some weighting matrices, and ***E*** and ***F*** are error matrices. Small means that it maximizes the coefficient of determination (R^2^) and minimizes the root mean square error of prediction (RMSEP), ensuring they effectively capture the underlying patterns in the *I* ×*K* that are most relevant for predicting the response variable vector ***Y***. In this study, *A* = 20 was chosen as the optimal number of latent variables. Predicting the content of the target response variable ($\hat{Y}$) for a new dataset, ($T_{\mathrm{pred}}$) is achieved using Equation (4):

(4) $$\hat{Y}=T_{\mathrm{pred}}\cdot \hat{C}$$

where $\hat{Y}$ represents the predicted content of the target response variable, $T_{\mathrm{pred}}$ represents the set of latent variables for these new observations, and $\hat{C}$ is the estimated matrix of regression coefficients. To ensure the reproducibility of our results, a random seed was set before using any modeling techniques. Additionally, we performed a random split of the dataset, selecting 70% for the training set and reserving 30% for the test set. This random data split allows one to train the PLSR model on one portion of the data and validate its performance on an independent dataset, thereby assessing its predictive accuracy. The prediction ability of the model was evaluated through several metrics, including the root mean square error of cross-validation (RMSECV), the root mean square error of prediction (RMSEP), correlation coefficients (R^2^), and the relative predictive deviation (RPD) for each model [32]. These metrics collectively assess the accuracy and reliability of our predictive models.

In addition to PLSR modeling, we used the expolinear function for visualization purposes. This function is defined by the Equation (5):

(5) $$y=\frac{cm}{rm}\cdot ln\left(1+e^{\left(rm\cdot \left(t-tb\right)\right)}\right)$$

where y represents the output variable (cannabinoid) and t denotes the input variable (wavelength spectra). Parameters cm,rm, and t are used for curve fitting. The ‘expolinear’ function was primarily employed to enhance the visual representation of our data.

## Figures and Tables

**Table 1:** Cannabinoid concentration of total cannabidiol (total CBD), total delta-9-tetrahydrocannabinol (total THC), and total cannabigerol (total CBG) with minimum, maximum, mean, and standard deviation (SD).

		Cannabinoid Concentration (%)			
Cannabinoid	*n*	Minimum	Maximum	Mean	SD
Total CBD	890	0.0	22.16	7.40	5.12
Total THC	890	0.0	16.33	2.49	3.88
Total CBG	890	0.0	13.76	0.66	1.34

**Table 2:** Cross-validation and prediction parameters of total cannabidiol (total CBD), total delta-9-tetrahydrocannabinol (total THC), and total cannabigerol (total CBG) concentrations through partial least-square regression (PLSR) models of raw observed data (Raw), standard normal variate (SNV), and Savitzky–Golay (SG) smoothing. The parameters include root mean square error of cross-validation (RMSECV), coefficient of determination for cross-validation (R^2^CV), root mean square error of prediction (RMSEP), coefficient of determination for prediction (R^2^P), and ratio of performance to deviation (RPD).

	Total CBD			Total THC			Total CBG		
Evaluation Criteria	Raw	SNV	SG Smoothing	Raw	SNV	SG Smoothing	Raw	SNV	SG Smoothing
RMSECV	2.399	2.282 *	2.405	1.583	1.524 *	1.557	0.584	0.627	0.577 *
R^2^CV	0.779	0.800 *	0.778	0.832	0.844 *	0.838	0.809	0.780	0.813 *
RMSEP	2.379	2.228 *	2.346	1.651	1.498 *	1.621	0.623 *	0.687	0.627
R^2^P	0.764	0.792 *	0.769	0.812	0.847 *	0.818	0.806 *	0.763	0.804
RPD	2.055	2.195 *	2.084	2.306	2.555 *	2.345	**2.267**	2.057	2.256

* Bold values indicate the best results.

## Data Availability

Data are contained within the article and Supplementary Materials.

## Supplementary Materials

The following supporting information can be downloaded at: https://www.mdpi.com/article/10.3390/plants13060833/s1. Figure S1: Observed versus predicted plot of the total delta-9-tetrahydrocannabinol (total THC) concentration for (a) raw data, (b) standard normal variate (SNV), and (c) Savitzky–Golay (SG) smoothing and total cannabigerol (total CBG) concentration for (d) raw data, (e) standard normal variate (SNV), and (f) Savitzky–Golay (SG) smoothing.
