# Supplementary material for: Non-Destructive Near-Infrared Technology for Efficient Cannabinoid Analysis in Cannabis Inflorescences
Source: Plants (Basel). 2024 Mar 14;13(6):833. doi: 10.3390/plants13060833 (PMC10975745; doi:10.3390/plants13060833)
Supplement: Supplementary file 1 [file plants-13-00833-s001.zip › plants-2903638-supplementary.pdf]

Supplementary Materials

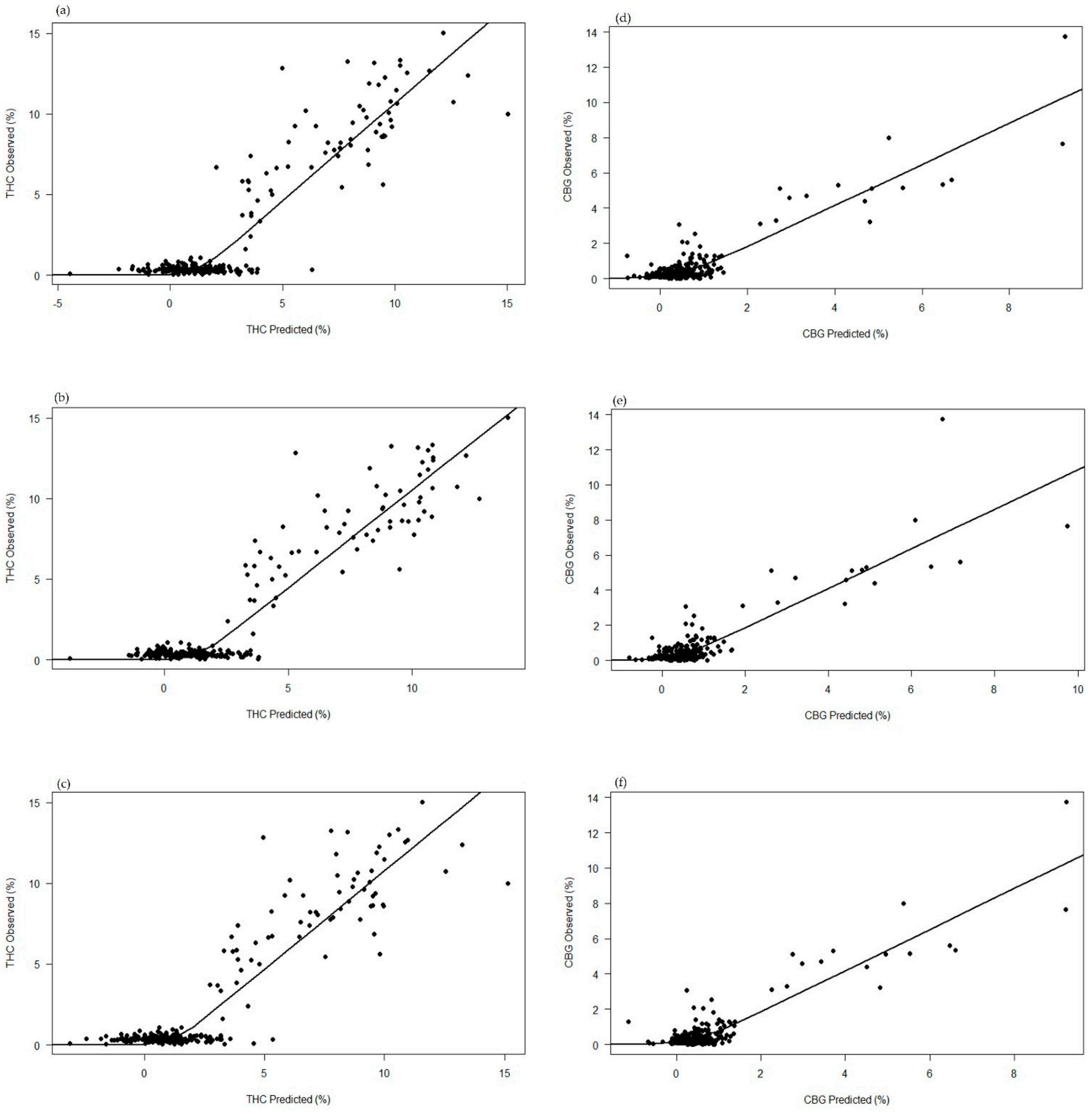

Figure S1. Observed versus predicted plot of the total delta-9-tetrahydrocannabinol (total THC) concentration for (a) raw data, (b) standard normal variate (SNV), and (c) Savitzky–Golay (SG) smoothing and total cannabigerol (total CBG) concentration for (d) raw data, (e) standard normal variate (SNV), and (f) Savitzky–Golay (SG) smoothing.
